# Tailoring the Use of Central Pancreatectomy Through Prediction Models for Major Morbidity and Postoperative Diabetes: International Retrospective Multicenter Study

**DOI:** 10.1097/SLA.0000000000006157

**Published:** 2023-12-11

**Authors:** Eduard Antonie van Bodegraven, Sanne Lof, Leia Jones, Béatrice Aussilhou, Gao Yong, Wei Jishu, Rosa Klotz, Dario Missael Rocha-Castellanos, Ippei Matsumato, Charles de Ponthaud, Kimitaka Tanaka, Esther Biesel, Emmanuele Kauffmann, Traian Dumitrascu, Yuichi Nagakawa, Pablo Martí-Cruchaga, Geert Roeyen, Alessandro Zerbi, Mara Goetz, Vincent E. de Meijer, Patrick Pessaux, Povilas Ignatavicius, Ihsan Ekin Demir, Mario Giuffrida, Bobby Tingstedt, Marco Vito Marino, Sotiris Mastoridis, Maximilian Brunner, Isabel Mora-Oliver, Cecilia Bortolato, Aisté Gulla, Thomas Apers, Hélène Hermand, Yusuke Mitsuka, Irinel Popescu, Ugo Boggi, Uwe Wittel, Satoshi Hirano, Sébastien Gaujoux, Keiko Kamei, Carlos Fernández-Del Castillo, Thilo Hackert, Jiang Kuirong, Miao Yi, Alain Sauvanet, Marc Besselink, Mohammad Abu Hilal, Safi Dokmak

**Affiliations:** * Amsterdam UMC, location University of Amsterdam, Department of Surgery, Amsterdam, The Netherlands; † Cancer Center Amsterdam, Department of Surgery, The Netherlands; ‡ Department of General Surgery, Istituto Ospedaliero Fondazione Poliambulanza, Brescia, Italy; § Department of HPB surgery and liver transplantation, Hospital of Beaujon, Clichy, France; ∥ The Pancreas Center of Nanjing Medical University, Department of Surgery, The First Affiliated Hospital with Nanjing Medical University, Nanjing, China; ¶ Department of General, Visceral and Transplantation Surgery, Heidelberg University Hospital, Heidelberg, Germany; # Department of Surgery, Massachusetts General Hospital and Harvard Medical School, Boston, MA, USA; ** Department of Surgery, Kindai University Faculty of Medicine, Osaka-sayama, Japan; †† Department of Digestive, Hepato-biliary-pancreatic and Liver Transplantation, Pitie-Salpetriere Hospital, AP-HP, Sorbonne University, Paris, France; ‡‡ Department of Gastroenterological Surgery II, Hokkaido University Faculty of Medicine, Hokkaido, Japan; §§ Medical Center-University of Freiburg, Department of General and Visceral Surgery, Freiburg, Germany; ∥∥ Division of General and Transplant Surgery, Department of Surgery, University of Pisa, Pisa; ¶¶ Center of General Surgery and Liver Transplant, Department of Surgery, Fundeni Clinical Institute, Carol Davila University of Medicine and Pharmacy, Bucharest, Romania; ## Department of Gastrointestinal and Pediatric Surgery Tokyo Medical University, Tokyo, Japan; *** Department of Surgery, Clínica Universitaria de Navarra, Pamplona, Navarra, Spain; ††† Department of HPB, Endocrine and Transplantation Surgery, Antwerp University Hospital, Belgium; ‡‡‡ Pancreatic Surgery Unit, Humanitas Clinical and Research Center-IRCCS, Department of Surgery, Rozzano, Milan, Italy; §§§ Department of General, Visceral and Thoracic Surgery, University Medical Center, Hamburg, Germany; ∥∥∥ Department of Surgery, University of Groningen and University Medical Center Groningen, Groningen, The Netherlands; ¶¶¶ Department of Visceral and Digestive surgery, Nouvel Hopital Civil, University Hospital, Strasbourg Institut Hospitalo-Universitaire de Strasbourg, Strasbourg, France; ### Department of Surgery, Lithuanian University of Health Sciences, Vilnius, Lithuania; **** Technical University of Munich, Department of Surgery, Germany; †††† Parma University Hospital-General Surgery Unit, Department of Surgery, Parma, Italy; ‡‡‡‡ Department of Surgery, University Hospital of Skane Lund, Lund, Sweden; §§§§ Department of Emergency and General Surgery, P. Giaccone, Hospital, University of Palermo, Italy; ∥∥∥∥ Department of Hepatobiliary and Pancreatic Surgery, Oxford University Hospitals NHS, Oxford, United Kingdom; ¶¶¶¶ Department of General and Visceral Surgery, University Hospital Erlangen, Friedrich Alexander University Erlangen-Nuremberg, Germany; #### Biochemical Research Institute, Department of Surgery, INCLICA, Hospital Clinico Universitario Valencia, Spain; ***** Department of Surgery, Ospedale dell’Angelo, Venice, Italy; ††††† Institute of Clinical Medicine, Vilnius University Faculty of Medicine, Department of Surgery, Vilnius, Lithuania; ‡‡‡‡‡ Department of General and Hepatopancreatobiliary Surgery and Liver Transplantation, Ghent University Hospital, Ghent, Belgium; §§§§§ Department of General, Visceral and Thoracic Surgery, University Hospital Hamburg-Eppendorf, Germany

**Keywords:** central pancreatectomy, complications, patient selection, risk model

## Abstract

**Objective:**

To develop a prediction model for major morbidity and endocrine dysfunction after central pancreatectomy (CP) which could help in tailoring the use of this procedure.

**Background:**

CP is a parenchyma-sparing alternative to distal pancreatectomy for symptomatic benign and premalignant tumors in the body and neck of the pancreas CP lowers the risk of new-onset diabetes and exocrine pancreatic insufficiency compared with distal pancreatectomy but it is thought to increase the risk of short-term complications, including postoperative pancreatic fistula (POPF).

**Methods:**

International multicenter retrospective cohort study including patients from 51 centers in 19 countries (2010–2021). The primary endpoint was major morbidity. Secondary endpoints included POPF grade B/C, endocrine dysfunction, and the use of pancreatic enzymes. Two risk models were designed for major morbidity and endocrine dysfunction utilizing multivariable logistic regression and internal and external validation.

**Results:**

A total of 838 patients after CP were included [301 (36%) minimally invasive] and major morbidity occurred in 248 (30%) patients, POPF B/C in 365 (44%), and 30-day mortality in 4 (1%). Endocrine dysfunction in 91 patients (11%) and use of pancreatic enzymes in 108 (12%). The risk model for major morbidity included male sex, age, Body Mass Index, and American Society of Anesthesiologists score ≥3. The model performed acceptably with an area under the curve of 0.72 (CI: 0.68–0.76). The risk model for endocrine dysfunction included higher Body Mass Index and male sex and performed well [area under the curve: 0.83 (CI: 0.77–0.89)].

**Conclusions:**

The proposed risk models help in tailoring the use of CP in patients with symptomatic benign and premalignant lesions in the body and neck of the pancreas (readily available through www.pancreascalculator.com).

Central pancreatectomy (CP) is a parenchyma-sparing alternative for distal pancreatectomy for symptomatic benign and premalignant lesions in the body and neck of the pancreas, such as pancreatic neuroendocrine tumors (pNETs), intraductal papillary mucinous neoplasms (IPMNs), and mucinous cystadenomas.^1,2^ Evidence on to what extent CP affects endocrine and exocrine function varies between negligible and 26% for endocrine dysfunction, and between 5% and 17% for exocrine dysfunction.^3–5^

CP requires both enteric anastomoses on the left pancreas, which is performed by pancreaticogastrostomy or pancreaticojejunostomy, and a pancreatic stump on the right pancreas.^3^ Therefore, pancreatic surgeons are typically reluctant to perform CP because of the presumed increased risk of complications, as compared with distal pancreatectomy. A recent systematic review, including 41 studies, found a postoperative pancreatic fistula (POPF) grade B/C rate of 23%, and an overall morbidity of 52% after CP.^3^ To date, however, no study has identified risk factors for major morbidity or POPF after CP.^6^ Furthermore, the feasibility of minimally invasive CP is not yet established and data on the safety of laparoscopic CP are mainly based on small cohort studies. Recently, a systematic review of 13 robot-assisted CP series, described a 42% rate of POPF B/C and a negligible diabetes rate.^7,8^

Overall, evidence on outcomes after CP is limited and mainly restricted to retrospective observational single-center studies. Multicenter, international studies are lacking and are needed to overcome the inherent bias in single-center studies. In the current study, we aim to aid individual patient selection for CP by identifying risk factors for major morbidity and endocrine dysfunction.

## METHODS

This is an international multicenter retrospective study on adult patients after CP. Centers were identified through: (1) a systematic literature search to identify centers that published a series exceeding 10 patients undergoing CP, (2) an invitation by the European-African Hepato-Pancreato-Biliary Association to all affiliated centers, and (3) an invitation by the International Consortium on minimally invasive pancreatic surgery to all affiliated centers. Interested centers received a questionnaire (GoogleSurvey, Mountain View) inquiring about their current implementation and opinion on minimally invasive and open CP.

This study was performed according to “Strengthening the Reporting of Observational Studies in Epidemiology” guidelines for cohort studies.^9^ The medical ethics review committee of the Amsterdam UMC waived the need for informed consent owing to the retrospective observational study design. Centers were invited to upload data of consecutive patients after open and minimally invasive CP for all indications [ie, benign and malignant (low-grade) tumors] between January 1, 2010 and December 31, 2021. Data were collected using Castor EDC (Castor, version 5.2.0).^10^ The center of origin was blinded and coded to guarantee anonymity on outcomes. Each participating center appointed one dedicated local study coordinator, responsible for the data collection and communication with the central study coordinator Eduard Antonie van Bodegraven. Each center received a password-protected database with the parameters of interest. All data were collected anonymously.

### Definitions

**FIGURE 1:**
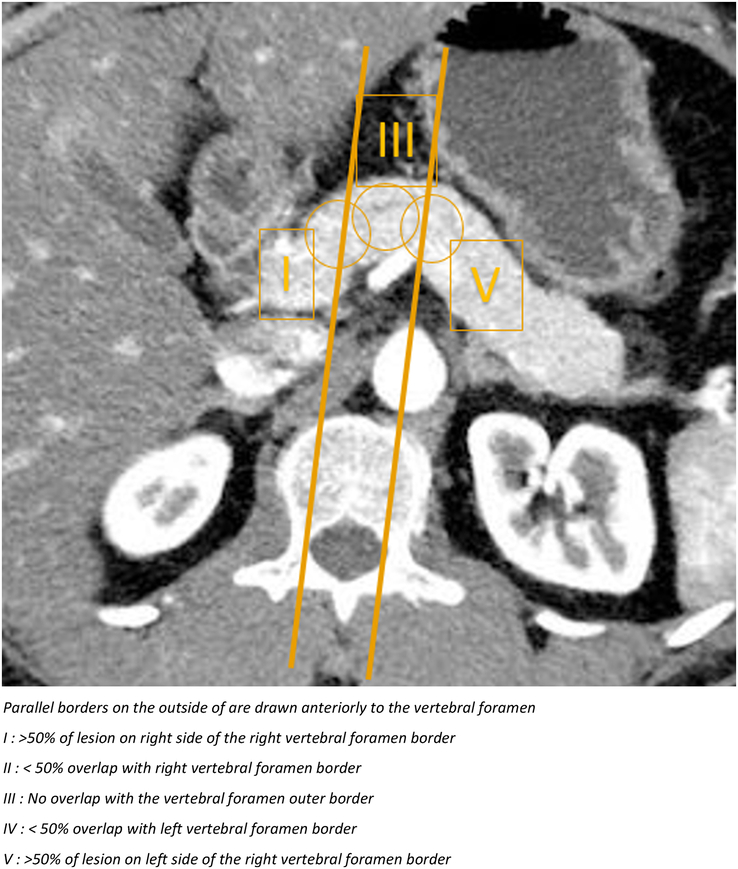
Preoperative location of pancreatic body lesions. Parallel borders on the outside are drawn anteriorly to the vertebral foramen. I: >50% of lesion on the right side of the right vertebral foramen border. II: <50% overlap with right vertebral foramen border. III: No overlap with the vertebral foramen outer border. IV: <50% overlap with left vertebral foramen border. V: >50% of lesion on the left side of the right vertebral foramen border.

Preoperative variables include age, sex, and Body Mass Index (BMI). Comorbidities include the American Society of Anesthesiologists (ASA) classification, diabetes, and past surgical history. Furthermore, information originating from the last preoperative computed tomography/magnetic resonance imaging scan (tumor size and location, distance to the main pancreatic duct, and vascular/other organ involvement) was noted. Pancreatic body lesions were defined as those with an anteroposterior overlap with the space between the articular processes of the vertebral body and are defined as in Figure 1 between body lesions type I to V. Preoperative diagnosis was defined as the working diagnosis, with all evidence retrieved in preoperative counseling. Minimally invasive procedures include both robot-assisted and laparoscopic CP. Conversion was defined as any resection started as a minimally invasive procedure (laparoscopic or robotic) that required laparotomy or hand assistance for reasons other than trocar placement or specimen extraction.^11^ Data on postoperative complications, readmissions, radiologic interventions, reoperations, and mortality were recorded up to 30 days postoperatively. Postoperative complications were classified using the Clavien-Dindo classification of surgical complications.^12^ Major morbidity was defined as Clavien-Dindo III or higher. Pancreatic-specific complications, that is, POPF, delayed gastric emptying, and postpancreatectomy hemorrhage, were collected according to the definitions of the International Study Group on Pancreatic Surgery.^13–15^

Endocrine dysfunction was defined as either new-onset diabetes or deterioration of preoperative diabetes which requires the start or increased dose of an antidiabetic drug. Exocrine pancreatic insufficiency (EPI) was defined as the need for pancreatic enzyme replacement therapy. Tumors were classified according to the latest World Health Organization classification. Resection margins were categorized according to the Royal College of Pathologists definition and classified into R0 (distance margin to tumor ≥1 mm), R1 (distance margin to tumor <1 mm), and R2 (macroscopically positive margin).^16^ Unexpected cancer rate was defined as a diagnosis of pancreatic ductal adenocarcinoma or any other malignant diagnosis with positive lymph nodes, with a nonmalignant working diagnosis.

### Endpoints

The primary endpoint was major morbidity, defined as the proportion of patients with Clavien-Dindo ≥III complications during admission and up to 30 days postoperative. Secondary endpoints include incidence of POPF International Study Group on Pancreatic Surgery grade B/C, conversion, blood loss, length of hospital stay, postoperative diabetes, pancreatic exocrine insufficiency, and in-hospital/30-day mortality.

### Statistical Analysis

Statistical analysis was performed using IBM SPSS Statistics for Windows version 26.0 (IBM). Student *t* test was used for comparison of normally distributed continuous variables, which are reported as mean (SD) values. Non-normally distributed variables were presented as median (interquartile range) values and compared using the Mann-Whitney *U* test. The normality of continuous variables was checked visually using histograms. Categorical variables were reported as counts with proportions, and analyzed with the χ^2^ or Fisher exact test, as appropriate. Preoperative variables with a *P* value <0.05 on univariable analysis were subjected to multivariable analysis, and a prediction model was developed using multivariable logistic regression modeling. The Akaike information criterion was used to optimize the number of covariates in the model.^17^ This implies the model with the smallest difference in predictive capacity is selected if this only leads to a marginal loss of predictive value compared with a more complex model. The model’s discriminative ability was assessed using the area under the receiver operating characteristic curve (area under the curve or concordance statistic).^18^ The goodness-of-fit was assessed using a calibration plot characterized by an intercept (ideal value: 0) and slope (ideal value: 1).^18^ Discrimination and calibration were assessed in an internal-external validation procedure according to current recommendations.^19^ This implied that the center group was left out once for validation of a model based on the data of the remaining cohorts. Smaller centers were grouped for this cross-validation procedure.^19^ An internal-external validation design is advised to combine the strength of external validation with the strength of prediction model development on all available data.^20^

## RESULTS

In total, 838 patients from 51 centers in 19 countries were included (Table 1). Overall, 77 centers completed the survey and 30 centers elucidated their considerations to perform no or few CP. In 53%, this was because the center had a low volume of potentially eligible patients, in 47%, this was because of a lack of experience, in 33%, this was because of a presumed or experienced high complication rate of CP, and in 16%, this was because it was unclear to the respondents what good indications for CP are. An annual volume of at least 5 CP procedures was observed in 2/51 (4%) centers.

**TABLE 1:** Baseline Characteristics

	n (%)
Sex (M)	331 (40)
Age, mean (SD)	52.53 (15.8)
BMI, mean (SD)	24.36 (4.3)
ASA ≥III	103 (12)
History of diabetes mellitus	135 (16)
History of abdominal surgery	199 (24)
Preoperative symptoms	341 (41)
Abdominal pain	191 (23)
Pancreatitis	33 (4)
Hypoglycemia	26 (3)
Other	22 (3)
Preoperative diagnosis	
pNET	197 (24)
IPMN	202 (24)
Solid pseudopapillary neoplasm	114 (14)
Mucinous cystic neoplasm	80 (10)
Chronic pancreatitis	43 (5)
Pancreatic ductal adenocarcinoma	31 (4)
Serous cystic neoplasm	34 (4)
Disrupted duct	14 (2)
Other	34 (4)
Unknown	73 (9)

### Baseline Characteristics

The mean age was 52 (15.8) years and the mean BMI was 24 (4.3) kg/m^2^. Most patients were females (60%), and 16% had a previous medical history of diabetes mellitus. The resected tumors had mostly been diagnosed incidentally (59%) and because of abdominal pain (23%). The main indications for surgery were pNETs (24%), IPMN (24%), solid pseudopapillary neoplasm (14%), and mucinous cystic neoplasm (10%), as displayed in Table 1. In 56% of the patients, the location of the tumor was in the corpus, and in 39%, in the neck of the pancreas.

### Operative

Among 838 patients undergoing CP, 537 (64%) were performed open and 301 (36%) minimally invasive. Among these minimally invasive procedures, 251 (30%) were laparoscopic and 50 (6%) were robot-assisted (Table 2). Laparoscopic CP was performed in 24 of the 51 (47%) centers, and robot-assisted CP in 13 of the 51 (25%) centers. The use of laparoscopic CP increased from 23% in 2010 to 2016 to 32% in 2017 to 2021, where the use of robot-assisted CP increased from 4% to 8%. The median operative time was 210 (150–270) minutes, the median intraoperative blood loss of 150 (50–260) mL, and the conversion rate of 4%. The pancreatic anastomosis was mostly a pancreaticojejunostomy (60%) and pancreaticogastrostomy (38%). The right-sided pancreatic stump was closed mainly by a stapler (56%) and most patients had intra-abdominal drainage (97%).

**TABLE 2:** Operative Details

	n (%)
Approach: open	537 (64)
Approach: laparoscopic	251 (30)
approach: robot-assisted	50 (6)
Operative time (min); median (IQR)	210 (150–270)
Interoperative blood loss (mL); median (IQR)	150 (50–260)
Conversion	11 (4)
Anastomosis: PJ	498 (60)
Anastomosis: PG	318 (38)
Pancreas texture: soft	636 (76)
Pancreas texture: hard/fibrotic	117 (14)
Pancreas texture: unknown	85 (10)
Stump closing: suture	373 (56)
Stump closing: staple	267 (40)
Splenic artery resection	12 (1)
Splenic vein resection	20 (2)
Drain placement	808 (97)

IQR indicates interquartile range; PG, pancreaticogastrostomy; PJ, pancreaticojejunostomy.

### Postoperative Morbidity

Postoperative morbidity is summarized in Table 3. Major morbidity (CD ≥III) was observed in 30% of patients and postoperative mortality in 1%. Overall morbidity was observed in 72% of patients and represented mainly POPF grade B/C (43%), postpancreatectomy hemorrhage grade B/C (11%), radiologic interventions (11%), and reoperations (6%). A total of 142 (16.9%) patients had unplanned intensive care admission. The median hospital stay was 14 days (interquartile range: 10–22), and 87 (13%) patients were readmitted after initial discharge.

**TABLE 3:** Postoperative Outcome

	n (%)
30 d morbidity	602 (72)
Major morbidity	248 (30)
POPF; grade B/C	365 (44)
Post pancreatectomy hemorrhage; grade B/C	95 (11)
Bile leak; grade B/C	5 (1)
Delayed gastric emptying; grade B/C	32 (4)
Chyle leak; grade B/C	9 (1)
Radiologic intervention	93 (11)
Reoperation	53 (6)
Highest Clavien-Dindo score; grade IIIa	139 (17)
Highest Clavien-Dindo score; grade IIIb–IVb	92 (11)
Highest Clavien-Dindo score; grade V	17 (2)
Hospital stay (d); median (IQR)	14 (10–22)
Intensive care admission ≥2 d	79 (23)
Duration of operative drainage (d); median (IQR)	13 (7–44)
30 d readmission	87 (13)
30 d mortality	4 (1)
Exocrine dysfunction	108 (13)
Endocrine dysfunction	92 (11)
New-onset diabetes mellitus	90 (11)
Worsening diabetes mellitus	2 (0)
Unexpected malignancy	18 (2)
Total lymph nodes retrieved; median (IQR)	0 (0–4)
No. of positive lymph nodes; median (IQR)	0 (0–1)

Major morbidity is defined as the Clavien-Dindo score ≥IIIa.

IQR indicates interquartile range.

### Pathology

Final histopathology showed pNET in 26% (n = 208), IPMN in 21% (n = 170), solid pseudopapillary neoplasm in 14% (n = 115), and mucinous cystic neoplasm in 14% (n = 110). Unexpected pancreatic cancer was found in 18 patients (2%), before surgery classified as IPMN (n = 8), pNET (n = 4), and solid pseudopapillary neoplasm (n = 3). At least one positive lymph node was present in 24 (3%) patients, in pancreatic ductal adenocarcinoma (n = 16) and pNET (n = 8).

### Endocrine Dysfunction and Exocrine Pancreatic Insufficiency

Endocrine dysfunction occurred in 92 patients (11%), which was defined as new-onset diabetes mellitus or worsening of diabetes mellitus. New-onset diabetes mellitus occurred in 90 patients (11%) and worsening diabetes mellitus by starting or intensifying antidiabetic treatment in 2 (0%). EPI was diagnosed in 108 patients (13%).^21^

### Risk Model Major Morbidity

The risk model to predict major morbidity included four variables: male sex [odds ratio (OR): 1.446 CI: 1.004–2.192], age (OR: 1.013 CI: 1.002–1.092 per year increase), BMI (OR: 1.060 CI: 1.011–1.112 per kg/m^2^), and ASA score ≥3 (OR: 1.714 CI: 1.045–3.075). The model performed acceptably with an area under a curve of 0.72 (CI: 0.68–0.76; Table 4). Three risk groups were identified: low-risk (<20%), intermediate-risk (20-50%), and high-risk (>50%) for major morbidity with 290 (35%), 442 (53%), and 106 (13%) patients, respectively.

**TABLE 4:** Risk Model for Major Morbidity After CP

Variable	OR	95% CI	*P*
Sex (M)	1.511	1.090–2.095	0.013
Age	1.022	1.012–1.033	<0.001
BMI	1.124	1.081–1.169	<0.001
ASA ≥3	3.694	2.318–5.886	<0.001
AUC (95% CI)	0.72 (0.68–0.76)		

$P=\frac{\exp[-5.296+0.022\left(\mathrm{age}\right)+0.413\left(\mathrm{male sex}\right)+0.117\left(\mathrm{BMI}\right)+1.307\left(\mathrm{ASA}\geq 3\right)]}{1+\exp[-5.296+0.022(\mathrm{age})+0.413(\mathrm{male sex})+0.117(\mathrm{BMI})+1.307\left(\mathrm{ASA}\geq 3\right)]}\;$

AUC indicates area under the curve.

### Risk Model Endocrine Dysfunction

In patients without preoperative diabetes mellitus, multivariate analysis revealed higher BMI (OR: 1.258 CI: 1.133–1.397) and male sex (OR: 5.268 CI: 2.080–13.340), as independent risk factors for postoperative endocrine dysfunction. The model performed well with an area under a curve of 0.83 (CI: 0.77–0.89; Table 5). Three risk groups were identified: negligible risk (<10%), low-risk (<10-20%), intermediate-risk (>20%) for postoperative diabetes mellitus with 509 (61%), 179 (21%), and 150 (18%) patients, respectively (both risk models are available at pancreascalculator.com, integrated as one model).

**TABLE 5:** Risk Model for Endocrine Dysfunction After CP

Variable	OR	95% CI	*P*
Sex (M)	5.268	2.080–13.340	<0.001
BMI	1.258	1.133–1.397	<0.001
AUC (95% CI)	0.83 (0.77–0.89)		

$P=\frac{\exp\left[-9.278+1.662(\mathrm{male sex})+0.230(\mathrm{BMI}\right)]}{1+\exp\left[-9.278+1.662(\mathrm{male sex})+0.230(\mathrm{BMI}\right)]}\;$

AUC indicates area under the curve.

## DISCUSSION

This largest international series to date on CP found a 30% rate of major morbidity, 43.5% POPF grade B/C, and 11% endocrine dysfunction in 838 patients from 51 centers in 19 countries. Two online available risk models use sex, age, BMI, and ASA score to predict major morbidity and endocrine dysfunction after CP and can be used to discuss and tailor the use of this procedure to the individual patient.

This is the first study to report on a risk model for major morbidity and endocrine dysfunction after CP and, therefore, these findings cannot be compared with other studies. Next, prospective studies should confirm the value of the proposed score. The risk model for endocrine dysfunction was based on patients without preoperative diabetes mellitus and included risk groups (<10%, 10%–20%, >20%). These risks should be weighed in relation to the 29% risk of new-onset diabetes after distal pancreactomy.^22^ Probably, when the risk of endocrine dysfunction after CP exceeds 20%, many surgeons and patients alike would choose a distal pancreatectomy rather than CP given the added risk of POPF B/C.

The outcomes reported in this study contrast with a 2022 systematic review, including 1004 patients after CP from 41 studies with a 23% rate of POPF B/C rate and 20% major morbidity.^3^ However, a 2018 systematic review comparing clinical outcomes of CP with distal pancreatectomy, found a POPF B/C rate of 35% after CP, which resembles the current findings.^23^ The discrepancy can possibly be explained by the more homogeneous data collection in the current study. Furthermore, the POPF grade B/C rate is high in the current study, with 43%, but the rate of Clavien-Dindo ≥III complications is 30%. This can be explained by the prolonged surgical drainage strategy which is used in some centers and may exceed the 21-day cutoff leading to POPF grade B, but may protect some patients from postoperative interventions.

The main reason to perform CP instead of distal pancreatectomy is the preservation of endocrine and exocrine function. Endocrine dysfunction after distal pancreatectomy occurs in 29% of patients according to a 2020 systematic review.^22,24^ New-onset diabetes mellitus occurred in 90 patients (11%) and worsening diabetes mellitus by starting or intensifying antidiabetic treatment in 2 (0%). Together, this was called endocrine dysfunction and both a higher BMI and male sex were found as risk factors. EPI occurred in 108 patients (13%) and was not associated with risk factors in multivariate analysis.

The presence of a preoperative dilated main pancreatic duct was not a risk factor for POPF after CP which is interesting as a dilated duct lowers the risk for POPF in case of pancreatic anastomosis (ie, pancreatoduodenectomy) but increases the risk for POPF after distal pancreatectomy.^6,25^ However, the current study design cannot be used to distinguish from which pancreatic transection plane (or both) a POPF occurred.

The results of the current study should be interpreted in light of some limitations. First, there was heterogeneity in reporting the postoperative endocrine dysfunction and EPI after CP. As only treated insufficiencies are taken in the analysis by the current study, subclinical insufficiencies may have been missed. Also, the severity of the dysfunctions was not taken into account as only the start or increase of antidiabetics or pancreatic enzyme replacement therapy was measured. Second, data on surgeons’ experience and learning curve were not available and, therefore, these factors and their correlation with short-term outcomes were not included in the analyses, although this could have influenced the results. Third, the lack of external validation of the currently proposed risk models is another limitation. However, all authors who published on CP in the last 30 years were invited to participate in the current study and, therefore, most patients available worldwide are in the current study and are an external validation cohort not likely to be realizable. An internal-external validation procedure, a type of cross-validation procedure, is recommended in the absence of an external validation cohort.^18,19^ Future, prospective studies should validate these risk scores.

Strengths of this study include its large size and multicenter international design with a focus on major morbidity and endocrine dysfunction providing the first risk models for CP. These models may aid both surgeons and patients in discussing the use of CP, balancing the benefits and downsides of this procedure in relation to distal pancreatectomy.

## CONCLUSIONS

This multicentre study provides the highest available level of evidence on CP and illustrates that CP should be considered in patients with benign or low-grade lesions in the body and neck of the pancreas as it lowers the risk of endocrine and exocrine insufficiency. With the risk calculator (readily available through www.pancreascalculator.com for major morbidity and postoperative diabetes) suitable patients can be selected.
